# Situation of Diabetes and Related Factors Among Qatari Adults: Findings From a Community-Based Survey

**DOI:** 10.2196/diabetes.7535

**Published:** 2017-05-03

**Authors:** Mohammed Al-Thani, Al-Anoud Al-Thani, Walaa Al-Chetachi, Shams Eldin Khalifa, Benjamin Vinodson, Badria Al-Malki, Ahmad Haj Bakri, Hammad Akram

**Affiliations:** 1 Ministry of Public Health Doha Qatar

**Keywords:** diabetes mellitus, obesity, public health, Qatar

## Abstract

**Background:**

Diabetes mellitus (DM) is a prominent public health problem in Qatar with one of the highest prevalence in the Gulf Cooperation Council region. Obesity continues to be a challenging public health problem in Qatar along with other social determinants contributing to the high DM prevalence.

**Objective:**

This paper examines the data from Qatar National STEPS survey (2012) to determine diabetes prevalence among Qatari adults and identify the effect of both generalized and central obesity on it. The article also describes the contribution of selected social and demographic factors on diabetes prevalence in Qatar.

**Methods:**

Secondary data analysis of 1471 Qatari adults (18-64 years) from STEP 3 component of the 2012 STEPS Survey was executed. Multivariate binary logistic regression analysis was carried out to assess the role of social and biomedical factors in the prevalence of DM.

**Results:**

Among participants, 18.97% (279/1471) of the study population had DM. Both generalized (OR 1.8, P=.005) and central obesity (OR 1.9, P<.001) were significantly associated with DM when adjusted for various respondent characteristics. Older age (P<.001), marital status of ever married (P<.001), and lower educational status (P=.01) were associated with DM. Hypertension (OR 1.5, P=.003 total cholesterol level ≥190 mg/dL (OR 2.2, P<.001) and triglyceride level ≥150 mg/dL (OR 3.6, P<.001) were significantly associated with DM among the study participants. Although family history of DM was significantly associated with development of DM (OR 1.7, P=.01), parental consanguinity was not associated with DM (OR 0.96, P=.80).

**Conclusions:**

The DM prevalence in Qatar seems to be highly associated with obesity; however, various additional population characteristics and comorbidity factors should also require attention and should be incorporated while developing intervention strategies.

## Introduction

Diabetes mellitus (DM) is one of the most costly preventable public health condition causing mortality and morbidity in millions of people globally [1]. It is diagnosed by a series of blood tests based on the raised blood glucose levels and is caused by either insufficient insulin production from pancreas (type 1 DM) or decreased ability of the body to use insulin (type 2 DM) [1]. Majority of DM cases are type 2 compared with type 1 and comprise 90% of people with DM around the world [1]. DM is known to increase the risks of cardiac, cerebrovascular, and kidney diseases and also contribute to complications associated with nerve damage such as diabetic retinopathy, foot ulcer, skin infections, and so on [2]. DM also doubles the risk of mortality among people with the disease than those without the disease [3].

According to the World Health Organization (WHO) estimates, in 2014 the global prevalence of DM in adults was 8.5%, and in 2012, mortality due to DM was 1.5 million worldwide [1]. Similarly, the prevalence of DM is also high in Arab region and has been identified to be a major disease burden in the Eastern Mediterranean Region countries where its prevalence ranges from 3.5% to 30% [4]. Increased body weight raises the risk of type 2 DM and other chronic conditions such as cardiovascular disease, musculoskeletal disorder, and cancers [1,5]. Since 1980, the obesity prevalence has doubled globally with over 600 million people estimated to be suffering from obesity [5]. Obesity is one of the contributory factors in developing insulin resistance which is the inability of the body to respond to insulin [6]. Body mass index (BMI) is considered a reliable parameter to assess the body weight and defined as weight (in kilograms) divided by the square of height (meters) of a person. The BMI ≥30 kg/m^2^ is classified as obesity [5]. Central obesity or wide waist circumference (WC) also increases the risk of developing type 2 DM sometimes even among individuals with normal or low BMI [7]. This is due to the fact that the increased degree and the duration of abdominal or body fat is associated with higher levels of insulin impairing hormones and chemicals in the body [7-9]. These excessive fat cells act like endocrine glands and therefore could eventually lead to DM if obesity is not controlled [7-9]. Lifestyle and cultural norms such as eating and exercise habits that may impact on body weight or fat content also influence the occurrence of DM in families [10].

The World Health Survey (WHS) in 2006 revealed that about 8% of Qatar population was diabetic which was higher than the worldwide diabetes prevalence of 5.1% [11]. Furthermore, the prevalence of diabetes among Qatari adults was higher (11.6%) compared with non-Qatari adult residents (6.6%) [12]. In 2011, the country’s estimated comparative diabetes prevalence for adults (aged between 20 and 79 years) was 20.1% ranking Qatar in top 5 among Arabic and non-Arabic speaking countries [3,4]. In 2012, 6% of deaths were related to DM in Qatar with higher proportion among Qatari nationals (9.9%) versus non-Qataris (4%) [12]. Type 2 DM is considered to be one of the leading causes of mortality due to non-communicable diseases (NCDs) in Qatar along with the cardiovascular disease and neoplasm [13]. Socioeconomic development in past decades has influenced the lifestyle of Qatari population which could be the reason that Qatar has one of the highest obesity prevalence in the Gulf Cooperation Council region [14,15]. As indicated in WHS 2006, a large proportion of population in Qatar was overweight (39%) and obese (32%). It was noted that obesity proportion was higher among Qatari nationals (40%) versus non-Qataris (28%) [11]. WHS 2006 report also showed that the likelihood of having DM was 1.5 times higher when BMI was greater than 30 [11].

This study examines the data from Qatar National STEPwise Survey (2012) to determine DM prevalence among Qatari adults and identify the effect of both generalized and central obesity on it. The STEPS survey is a standardized approach to collect population level data pertaining to the NCD including DM and the related risk factors [16,17]. The aim of this study is to also describe the contribution of selected social and demographic factors on DM prevalence in Qatar. This area has not been extensively explored and published before; hence, the findings from this study could help in designing effective public health techniques and interventions to reduce DM-related mortality and morbidity in the county.

## Methods

### Sampling

The secondary data from WHO-based Qatar National STEPwise Survey for chronic diseases and risk factors were utilized for this particular study [16]. The STEPS was implemented between March and May of 2012 in which 2496 Qatari nationals aged 18-64 years were randomly selected with a response rate of 88%. A multistage cluster sampling design was used for the survey. Using a two-stage sampling design, a total of 96 primary sampling units (PSUs) were selected at the first stage. In the second stage, 30 households were selected from each selected PSU by simple systematic sampling.

The DM status by obesity and sociodemographic factors was examined among 1471 Qatari adults aged 18-64 years and who participated in the STEP 3 survey tool. The response rate for STEP 3 component was about 60% [16]. STEPwise consists of three survey tools: STEP 1 is for gathering demographic and behavioral risk factors information; STEP 2 is for collecting physical measurements such as weight, height, WC and blood pressure (BP); and STEP 3 is for taking blood samples for biochemical measurements: fasting blood glucose level (FBG), and lipid profile, respectively [16,17]. The STEPS methodology provided a sample which was representative of all Qatari nationals. The sampling design took into account the WHO STEPS formula for calculating the sample size, as specified in the STEPS guidelines for participating countries and considered as multistage, systemic random sampling [16,17].

Data to assess obesity in STEP 3 participants were analyzed using the STEPS methodology [16,17]. BMI and WC cutoff values were determined as per WHO recommendations, that is, BMI ≥30 kg/m^2^ denoted generalized obesity and WC ≥102 in males and ≥88 in females represented central obesity [8,18]. Data for pregnant women were removed from the analysis while performing body weight and WC calculations.

Cutoff values for FBG, lipid profile, and BP were determined in accordance with the WHO STEPS Guidelines, that is ≥190 mg/dL of total cholesterol, ≥150 mg/dL triglyceride level, <40 mg/dL and <50 mg/dL of high-density lipoproteins (HDL) in men and women, respectively, and ≥110 mg/dL of FBG were considered unfavorable [16,17]. The participants who had FBG levels ≥110 mg/dL and/or were taking insulin or other prescribed medications to control their DM (regardless of their FBG results) were considered diabetics. The participants with systolic BP of ≥140 mmHg and/or diastolic BP ≥90 mmHg or who were currently on medications for raised BP were considered hypertensive [16].

### Data Analysis

Statistical analysis was performed using IBM SPSS statistics version 20.0 for Windows. All categorical variables were presented as frequencies and percentages. Study outcomes were assessed using Fisher exact test or chi-square test with Yates correction for discrete variables appropriately. The Kolmogorov-Smirnov test was used for checking normality. Continuous variable such as FBG, BMI, WC were presented as geometric mean and 95% CI. Independent sample *t* test was used to compare means across two different groups. Odds ratio (OR) and 95% CI was computed using multivariate logistic regression, which identifies the degree of the association between DM and various factors. In the crude model, no adjustments were made, whereas age and gender were controlled in the first model. Second model was adjusted for age, gender, and sociodemographic indicators such as marital status, education, and smoking status. In addition to the factors included in model 2, family history of DM (among parents, siblings, and/or children) and parental consanguinity variables were controlled in the final model 3. The sensitivity, specificity, positive predictive values, and negative predictive values were calculated using family history of DM, parental consanguinity and obesity variables and their impact on diabetic status of participants. This was used to test the ability of factors to correctly identify respondents with and without DM. The *P* value <.05 was considered statistically significant.

## Results

### Sample Characteristics

The female-to-male ratio among the total surveyed population (N=1471) was 1.7 (62.5% women and 37.5% men). The overall mean age of participants was 38.5 years with higher mean age (45.8 years) for diabetics (*P*<.001). Almost 80.35% (n=1182) of the study participants were currently or previously married. In relation to the level of education, the results showed that over one-third of the participants completed their college or university education (34.9%, n=513). Overall parental consanguinity was reported by 36% (n=528) of the respondents and 69.14% (n=1017) stated that they had a family history of DM. In accordance with the study criteria, 19% (n=279) of participants had DM with women comprising 60.2% (n=168) of diabetics. By gender, 20.1% (n=111) of male and 18.3% (n=168) of female participants had DM. About 34.1% (n=158) of the participants aged 45-64 years and 12% (n=121) of the participants aged 18-44 years had DM. Mean WC for males was 102 cm, higher among diabetics (108 cm) compared with nondiabetics (100.8 cm; *P*=.01). Mean BMI was also high among diabetics (*P*<.01). Mean HDL level was significantly lower among diabetic women compared with nondiabetic (*P*=.04). Mean total cholesterol (*P*=.002) and triglyceride levels (*P*<.01) were higher among diabetic participants. Both, mean systolic and diastolic BP readings were higher among diabetics (*P*<.01). The characteristics of participants are shown in Table 1.

**Table 1:** Study participants' characteristics by diabetes status. Geometric means are presented with 95% CI. Age is presented with SD. Pregnant women are excluded from the BMI and WC calculations.

Parameters				Categories		Total, N (%)		Diabetic, n (%)		Normal, n (%)		*P*
Overall sample				N		1471 (100.0)		279 (19.0)		1192 (81.0)		
				Mean (SD)		38.5 (12.2)		45.8 (11.7)		36.7 (11.7)		<.001
Age				18-44 years		1008 (68.5)		121 (43.4)		887 (74.4)		<.001
				45-64 years		463 (31.5)		158 (56.6)		305 (25.6)		
Gender				Male		552 (37.5)		111 (39.8)		441 (37.0)		.38
			Female		919 (62.5)		168 (60.2)		751 (63.0)		
BMI (kg/m^2^)				Mean (95% CI)		29.4 (29.0-29.8)		31.8 (30.9-32.7)		28.8 (28.4-29.3)		<.001
				Lean		39 (2.7)		1 (0.4)		38 (3.3)		<.001
				Normal		315 (22.2)		34 (12.5)		281 (24.4)		
				Overweight		403 (28.3)		75 (27.6)		328 (28.5)		
				Obese		665 (46.8)		162 (59.6)		503 (43.7)		
**Waist circumference (cm)**															
	Male		Mean (95% CI)		102.2 (100.0-104.5)		108.0 (103.1-113.2)		100.8 (98.3-103.3)		.01	
				Central obesity (WC ≥102 cm)		268 (49.3)		71 (64.5)		197 (45.4)		<.001	
				Normal (WC <102 cm)		276 (50.7)		39 (35.5)		237 (54.6)			
	Female		Mean (95% CI)		97.8 (94.7-100.8)		102.1 (98.1-106.1)		96.8 (93.3-100.5)		.20	
				Central obesity (WC ≥88 cm)		509 (60.0)		127 (79.9)		382 (55.4)		<.001	
				Normal (WC <88 cm)		340 (40.0)		32 (20.1)		308 (44.6)			
Education level				Secondary or less/no formal education		957 (65.1)		200 (71.7)		757 (63.6)		.01
				College/university/PG		513 (34.9)		79 (28.3)		434 (36.4)		
Marital status				Ever married		1182 (80.4)		256 (91.8)		926 (77.7)		<.001
				Never married		289 (19.6)		23 (8.2)		266 (22.3)		
Parental consanguinity				Yes		528 (35.9)		97 (34.8)		431 (36.2)		.66
				No		943 (64.1)		182 (65.2)		761 (63.8)		
Family history of DM				Yes		1017 (69.1)		208 (74.6)		809 (67.9)		.03
				No		454 (30.9)		71 (25.4)		383 (32.1)		
Mean fasting blood glucose (95% CI)				Overall		90.3 (88.8-92.0)		149.2 (143.5-155.1)		80.3 (79.6-81.0)		<.001
				Men		90.2 (87.8-92.7)		148.8 (140.1-158.2)		79.6 (78.3-80.8)		<.001
				Women		90.3 (88.5-92.1)		149.4 (142.0-157.1)		80.7 (79.8-81.6)		<.001
Current smoker				Yes		197 (13.4)		35 (12.5)		162 (13.6)		.64
				No		1274 (86.6)		244 (87.5)		1030 (86.4)		
Smoking status				Daily		179 (12.2)		31 (11.1)		148 (12.4)		.82
				Nondaily		18 (1.2)		4 (1.4)		14 (1.2)		
				Past smoker		53 (3.6)		12 (4.3)		41 (3.4)		
				Never smoker		1221 (83.0)		232 (83.2)		989 (83.0)		
Hypertension				Mean systolic blood pressure (SBP), mmHg (95% CI)		118.3 (117.4-119.2)		127.4 (125.1-129.8)		116.2 (115.3-117.1)		<.001
				Mean diastolic blood pressure (DBP), mmHg		79.0 (78.4-79.5)		83.0 (81.8-84.2)		78.0 (77.4-78.6)		<.001
				SBP ≥140/DBP ≥90 mmHg or on medication		540 (37.0)		150 (54.0)		390 (33.1)		<.001
				Normal		918 (63.0)		128 (46.0)		790 (66.9)		
Total cholesterol (mg/dL)				Mean (95% CI)		160.4 (158.6-162.2)		166.6 (161.8-171.4)		159.0 (157.0-160.9)		<.01
				≥190 mg/dL		381 (26.0)		124 (44.6)		257 (21.7)		<.001
				<190 mg/dL		1083 (74.0)		154 (55.4)		929 (78.3)		
**HDL (mg/dL)**														
	Male		Mean (95% CI)		39.7 (38.5-40.9)		38.8 (36.4-41.4)		39.9 (38.5-41.3)			.47
				<40 (mg/dL)		264 (47.8)		59 (53.2)		205 (46.5)		.21
				≥40 (mg/dL)		288 (52.2)		52 (46.8)		236 (53.5)		
	Female		Mean (95% CI)		54.5 (53.4-55.6)		52.0 (49.4-54.8)		55.1 (53.8-56.4)			.04
				<50 (mg/dL)		341 (37.4)		73 (43.7)		268 (36.0)		.06
				≥50 (mg/dL)		571 (62.6)		94 (56.3)		477 (64.0)		
Triglyceride (mg/dL)				Mean (95% CI)		96.1 (93.8-98.5)		122.9 (115.7-130.7)		90.7 (88.4-93.1)		<.001
				≥150 (mg/dL)		257 (17.7)		103 (37.2)		154 (13.1)		<.001
				<150 (mg/dL)		1197 (82.3)		174 (62.8)		1023 (86.9)		
LDL (mg/dL)				Mean (95% CI)		89.1 (87.6-90.5)		89.5 (86.1-93.1)		88.9 (87.3-90.5)		.75
				≥130 (mg/dL)		144 (10.8)		34 (12.8)		110 (10.3)		.24
				<130 (mg/dL)		1188 (89.2)		232 (87.2)		956 (89.7)		

### Crude Analysis

Crude analysis showed that the older age group (45-64) had 3.8 time odds of having DM compared with younger age (18-44) group (95% CI 2.9-4.9, *P*<.01). By gender, male participants showed 1.1 time odds of having DM compared with females, but this relationship was not statistically significant (95% CI 0.86-1.5, *P*=.38). Secondary or lower educational status was found to be associated with development of DM among study participants (OR 1.45, 95% CI 1.1-1.9, *P*=.01). Participants who were currently or previously married also had higher odds of having DM compared to the ones who were never married (OR 3.2, 95% CI 2.0-5.0, *P*<.01). The detailed crude associations of predictors and participant categories are shown in Table 2.

**Table 2:** Relationship (crude) of diabetes between participant parameters and categories. Odds ratios (OR) and 95% CI were estimated using logistic regression models. Model 0: crude odds ratio.

Parameters			Categories		Crude OR (95% CI)			
Predictors					OR (95% CI)		*P*	
Age			18-44		Reference			
			45-64		3.8 (2.9-4.9)		<.001	
Gender			Male		1.1 (0.86-1.5)		.38	
			Female		Reference			
Marital status			Ever married		3.2 (2.0-5.0)		<.001	
			Never married		Reference			
Highest level of education			Secondary or less/no formal education		1.45 (1.09-1.9)		.01	
			College/University/PG		Reference			
Parental consanguinity			Yes		0.94 (0.72-1.2)		.66	
			No		Reference			
Family history of DM			Yes		1.4 (1.03-1.8)		.03	
			No		Reference			
Smoking status			Daily		0.89 (0.59-1.3)		.59	
			Nondaily		1.22 (0.39-3.7)		.73	
			Past smoker		1.24 (0.64-2.4)		.51	
			Never smoker		Reference			
BMI (kg/m^2^)			Lean		0.21 (0.03-1.6)		.14	
			Normal		Reference			
			Overweight		1.9 (1.2-2.9)		.004	
			Obese		2.6 (1.8-3.9)		<.001	
**Waist circumference (cm)**									
	Male		≥102 cm		2.2 (1.4-3.4)			<.001	
			<102 cm		Reference		
	Female		≥88 cm		3.2 (2.1-4.8)			<.001	
			<88 cm		Reference		
Blood pressure (BP, mmHg)			Raised BP or currently on medication		2.3 (1.8-3.1)			<.001	
			Normal		Reference			
Total cholesterol (mg/dL)			≥190 mg/dL		2.9 (2.2-3.8)		<.001	
			<190 mg/dL		Reference			
**HDL (mg/dL)**										
	Male		<40 (mg/dL)		1.3 (0.86-1.9)		.21	
			≥40 (mg/dL)		Reference			
	Female		<50 (mg/dL)		1.4 (0.98-1.9)		.06	
			≥50 (mg/dL)		Reference			
Triglyceride (mg/dL)			≥150 (mg/dL)		3.9 (2.9-5.3)		<.001	
			<150 (mg/dL)		Reference			
LDL (mg/dL)			≥130 (mg/dL)		1.3 (0.84-1.9)		.25	
			<130 (mg/dL)		Reference			

### Multivariate Analysis

Multivariate logistic regression analysis revealed that generalized obesity was significantly associated with DM (OR 1.8, 95% CI 1.2-2.8, *P*=.005); however by gender, this relationship was only significant among females (OR 2.2, 95% CI 1.2-4.0, *P*=.009) versus males (OR 1.4, 95% CI 0.78-2.7, *P*=.23). Central obesity was found to also be associated with DM in overall sample (OR 1.9, 95% CI 1.4-2.6, *P*<.01), among males (OR 1.8, 95% CI 1.1-2.9, *P*=.007) and as well as among females (OR 2.0, 95% CI 1.2-3.1, *P*=.003; Table 3). Hypertension (OR 1.5, 95% CI 1.1-2.0, *P*=.003), total cholesterol level ≥190 mg/dL (OR 2.2, 95% CI 1.6-3.0, *P*<.01) and triglyceride level ≥150 mg/dL (OR 3.6, 95% CI 2.6-4.9, *P*<.01) were significantly associated with DM among study participants (Table 3). HDL and LDL levels did not show a significant relationship in DM causation (Table 3).

Family history of DM was significantly associated with DM (OR 1.7, 95% CI 1.2-2.3, *P*=.001; Table 3). Parental consanguinity did not have any impact on diabetic status (OR 0.96, 95% CI 0.72-1.3, *P*=.77; Table 3).

Including obesity parameters in the relationship between consanguinity and family history with obesity, respectively (not shown in the table), showed that the family history of DM in presence of generalized obesity was not statistically significant (OR 1.3, 95% CI 0.95-1.7, *P*=.09) in having diabetic status; however, it was slightly significant for central obesity (OR 1.4, 95% CI 1.0-1.8, *P*=.05). Parental consanguinity had no influence on development of DM among participants with generalized (OR 0.97, 95% CI 0.73-1.3, *P*=.86) as well as central obesity (OR 1.02, 95% CI 0.77-1.4, *P*=.85).

**Table 3:** Relationship of diabetes between participant parameters and categories, multivariate logistics regression models. Odds ratios (95% CI) were estimated using multivariate logistic regression models.

Parameters		Categories	Multivariate models					
			Model 1^a^		Model 2^b^		Model 3^c^	
Predictors			OR (95% CI)	*P*	OR (95% CI)	*P*	OR (95% CI)	*P*
BMI (kg/m^2^) excluding pregnant		Lean	0.29 (0.04-2.2)	.23	0.35 (0.04-2.6)	.31	0.36 (0.04-2.7)	.33
		Normal	Reference		Reference		Reference	
		Over weight	1.5 (0.97-2.4)	.06	1.4 (0.92-2.3)	.1	1.4 (0.91-2.3)	.12
		Obese	2.1 (1.4-3.2)	<.001	1.9 (1.2-2.9)	.002	1.8 (1.2-2.8)	.005
**Waist circumference (cm)** (no gender, no pregnant)							
	Male	≥102 cm	2.0 (1.3-3.2)	.002	1.9 (1.2-2.9)	.006	1.8 (1.1-2.9)	.007
		<102 cm	Reference		Reference		Reference	
	Female	≥88 cm	2.3 (1.5-3.5)	<.001	2.1 (1.3-3.2)	.002	2.0 (1.2-3.1)	.003
		<88 cm	Reference		Reference		Reference	
Blood pressure (BP, mmHg)		Raised BP or currently on medication	1.7 (1.3-2.2)	<.001	1.6 (1.2-2.1)	.001	1.5 (1.1-2.0)	.003
		Normal	Reference		Reference		Reference	
Total cholesterol (mg/dL)		≥190 mg/dL	2.3 (1.8-3.2)	<.001	2.3 (1.7-3.1)	<.001	2.2 (1.6-3.0)	<.001
		<190 mg/dL	Reference		Reference		Reference	
**HDL (mg/dL)**							
	Male	<40 (mg/dL)	1.3 (0.87-2.1)	.18	1.32 (0.85-2.1)	.21	1.28 (0.82-2.0)	.27
		≥40 (mg/dL)	Reference		Reference		Reference	
	Female	<50 (mg/dL)	1.3 (0.93-1.9)	.11	1.29 (0.91-1.8)	.15	1.3 (0.89-1.8)	.16
		≥50 (mg/dL)	Reference		Reference		Reference	
Triglyceride (mg/dL)		≥150 (mg/dL)	3.9 (2.8-5.3)	<.01	3.7 (2.7-5.1)	<.01	3.6 (2.6-4.9)	<.001
		<150 (mg/dL)	Reference		Reference		Reference	
LDL (mg/dL)		≥130 (mg/dL)	1.1 (0.75-1.7)	.54	1.1 (0.73-1.7)	.6	1.1 (0.71-1.7)	.66
		<130 (mg/dL)	Reference		Reference		Reference	
Family history of DM		Yes	1.6 (1.1-2.1)	.004	1.7 (1.2-2.3)	.001		
		No	Reference		Reference			
Parental consanguinity		Yes	0.97 (0.73-1.3)	.86	0.96 (0.72-1.3)	.77		
		No	Reference		Reference			

^a^Model 1: adjusted for age and gender.

^b^Model 2: adjusted for age, gender, sociodemographic indicators (marital status, education, smoking status).

^c^Model 3: adjusted for age, gender, sociodemographic indicators, family history, and consanguinity.

### Family History, Consanguinity, and Obesity as a Screening Tool

Using family history as a screening tool, the family history of DM identified 74.6% of participants who had DM. For obesity (generalized), 59.6% of participants were identified to have DM. Family history and obesity together identified 46% diabetics. This means that family history is a better indicator of DM among participants compared with obesity or other combinations including consanguinity (Table 4). Positive predictive value which identify the participants who truly had disease during screening, showed that the family history of DM predicted DM among 20.4% of participants, while obesity predicted DM among 24.4% of participants. Obesity and family history together increased the prediction of DM to 25.7 %. Consanguinity alone increased the prediction of DM to 18.3%, but consanguinity with obesity increased it to 23.7% and with family history and obesity to 25.1% (Table 4).

**Table 4:** Sensitivity, specificity, positive and negative predictive values of family history of diabetes, consanguinity, obesity and all their possible combinations.

Selected social characteristics	Sensitivity % (95% CI)	Specificity % (95% CI)	Positive predictive value % (95% CI)	Negative predictive value % (95% CI)
Family history of DM	74.6 (69.0-79.6)	32.1 (29.5-34.9)	20.4 (19.2-21.7)	84.4 (81.3-87.0)
Consanguinity	34.8 (29.2-40.7)	63.8 (61.0-66.6)	18.3 (15.8-21.2)	80.7 (79.2-82.1)
Obesity	59.6 (53.5-65.4)	56.3 (53.4-59.1)	24.4 (22.2-26.6)	85.5 (83.6-87.3)
Family history of DM with obesity	46.0 (39.9-52.1)	68.6 (65.8-71.3)	25.7 (22.9-28.8)	84.3 (82.7-85.8)
Consanguinity with obesity	20.2 (15.6-25.5)	84.6 (82.4-86.6)	23.7 (19.1-28.9)	81.8 (80.8-82.7)
Consanguinity with family history of DM	26.5 (21.4-32.1)	75.1 (72.5-77.5)	19.9 (16.7-23.7)	81.4 (80.2-82.5)
Consanguinity and family history of DM with Obesity.	16.2 (12.0-21.1)	88.6 (86.6-90.4)	25.1 (19.7-31.5)	81.7 (80.9-82.5)

## Discussion

### Principal Findings

In summary, the results of this study support the fact that the family history of DM, older age, high WC, high BMI, hypertension, dyslipidemia, lower educational status, and marital status (ever married) have significant relationship with DM and are consistent with the findings from other studies [19-22]. The individuals with higher educational status tend to avoid unhealthy behaviors such as physical inactivity, alcohol abuse, smoking, and so on. [23]. This is usually coupled with the knowledge and circumstances favoring better understanding of health needs and easier access to the health care services [23,24]. Even though family history of DM is a reasonable indicator of DM in this study and supports the fact that the genetics may have a strong influence on burden of disease among participants; however, the impact of other social, environmental, and health factors cannot be ignored

DM prevalence is a result of complex interaction between personal, social, economic, and environmental factors in a geographical region. This study demonstrates DM as an important public health challenge in Qatar somewhat similar to the other countries in the region [11,14,15]. Overall, 19% of sample had DM with a higher frequency among women compared with men (Table 1). Furthermore, the STEPwise data shows that Qatar has a higher prevalence of obesity especially among women [16,25]. This gender distribution is similar to a study conducted in the Saudi Arabia (2012) in which female participants had higher obesity compared with men [26]. The overall DM prevalence was also similar in the Saudi Arabia study (21.5%); however, a higher percentage of DM was observed among males compared with females [26]. Even though 60.2% of participants among diabetics group were females, it is important to mention that the percentage of having DM among male participants (out of all male participants) was slightly higher than females.

While examining the impact of obesity on DM, both generalized and central types obesity were found to be significantly associated with DM in this study. Central obesity among females had slightly higher odds (OR 2.0) of having DM versus males (OR 1.8; Table.3). According to the 2001 Korea National Health and Nutrition Examination Survey (KNHANES), WC and BMI both were identified as a risk factor for DM in females and WC was also associated with DM in males [27]. A meta-analysis based on 18 prospective cohort studies showed that the obese and overweight individuals were at 7 and 3 times higher risk of developing DM, respectively, when compared with those with normal weight [28]. The same study also showed that females with obesity were relatively at higher risk of developing DM compared with males [28].

In this study, the family history of DM was found among about 70% of STEP 3 survey participants. The data also showed that around 20% of survey participants who had family history of DM also had DM which constitutes 74.6% of identified 279 diabetics in the sample of this study (Table 1). The high prevalence of DM with family history coincides with the findings from other studies [19,29-31]. The study also revealed that hypertension was a risk factor in the development of DM among participants which is consistent with the findings from a study based on UK Clinical Practice Research Datalink [32].

### Strengths and Limitations

In this study, using data from a national population based survey was a main strength [16]. The survey is based on the WHO-STEPS protocol and considered a standardized tool [17]. The STEPS survey methodology provides a representative sample from a target population [16,17]. Furthermore, the DM status was determined by using subjective as well as objective (bio-chemical) responses/outcomes from the survey data to avoid any missing individuals with DM, for example, the ones who were previously diagnosed to have DM and had FBG levels less than cut off point. Like any other cross-sectional study, STEPS survey in Qatar also faced issues pertaining to the response rates, recall bias and associated misclassification. One of the study limitation is that the findings may not be directly comparable with other studies from different geographical regions, mainly due to the differences in methodology; however, it can be compared with the findings that were obtained using STEPS or similar methodology.

### Conclusions

According to this study, the central and generalized obesity both have an impact on the DM prevalence among Qatari adults. Furthermore, social and behavioral factors seem to have an influence on DM prevalence. In general, DM and obesity together are a major problem in the State of Qatar that requires evidence-based strategies to reduce associated morbidity and premature death. The results of this study might help public health and medical professionals in planning and implementing effective and sustainable interventions.

## Abbreviations

BMI: Body mass index
BP: Blood pressure
DM: Diabetes mellitus
FBG: Fasting blood glucose
NCDs: Noncommunicable diseases
PSUs: Primary sampling units
STEPS: STEPwise survey
WC: Waist circumference
WHO: World Health Organization
WHS: World health survey
